# Vitamin B5 supplementation enhances intestinal development and alters microbes in weaned piglets

**DOI:** 10.1080/10495398.2024.2335340

**Published:** 2024-04-08

**Authors:** Xin Wang, Yan Qin, Jianzhong Li, Pengfei Huang, Yali Li, Jing Huang, Qiye Wang, Huansheng Yang

**Affiliations:** a Hunan Provincial Key Laboratory of Animal Intestinal Function and Regulation, Hunan International Joint Laboratory of Animal Intestinal Ecology and Health, Laboratory of Animal Nutrition and Human Health, College of Life Sciences, Hunan Normal University, Changsha, Hunan, China; b Key Laboratory of Agro-ecological Processes in Subtropical Region, Hunan Provincial Engineering Research Center of Healthy Livestock, Scientific Observing and Experimental Station of Animal Nutrition and Feed Science in South-Central, Ministry of Agriculture, Institute of Subtropical Agriculture, Chinese Academy of Sciences, Changsha, Hunan, China

**Keywords:** Intestinal development, gut microbiota, short-chain fatty acid, vitamin B5, weaned piglet

## Abstract

This study explored the effects of different vitamin B5 (VB5) levels on intestinal growth and function of weaned piglets. Twenty-one piglets (7.20 ± 1.11 kg) were included in a 28-day feeding trial with three treatments, including 0 mg/kg (L-VB5), 10 mg/kg (Control) and 50 mg/kg (H-VB5) of VB5 supplement. The results showed that: Large intestine weight/body weight was the highest in H-VB5 group, Control and H-VB5 groups had significantly higher villus height and villus height/crypt depth than the L-VB5 in the ileum (*p* < .05). Goblet cells (ileal crypt) and endocrine cells (ileal villus) significantly increased in Control and H-VB5 (*p* < .05). The H-VB5 group exhibited significantly higher levels of ki67 and crypt depth in the cecum and colon, colonic goblet cells and endocrine cells were both rising considerably (*p* < .05). Isobutyric acid and isovaleric acid were significantly reduced in the H-VB5 group (*p* < .05), and there was a decreasing trend in butyric acid (*p* = .073). At the genus level, the relative abundance of harmful bacteria such as *Clostridium_Sensu_Structo_1 Strecto_1*, *Terrisporbacter* and *Streptococcus* decreased significantly and the relative abundance of beneficial bacteria *Turicibacter* increased significantly in H-VB5 group (*p* < .05). Overall, the addition of 50 mg/kg VB5 primarily enhanced the morphological structure, cell proliferation and differentiation of the ileum, cecum and colon. It also had a significant impact on the gut microbiota and short-chain fatty acids.

## Introduction

Pigs in all phases of growth and development need a healthy digestive system to maintain their general metabolism, physiology, health condition and performance.^1^ Previous research has shown that even in the absence of disease, the pig’s gastrointestinal tract can be compromised.^2^ Low feed intake after weaning, for example, can result in piglets’ intestinal lumens lacking adequate nutrients.^3^ Furthermore, numerous studies have demonstrated that the presence of nutrients in the lumen after weaning has a direct impact on the maintenance of intestinal structure and function.^4^^,^^5^ Piglet weaning also results in significant changes in the structure and function of the gastrointestinal tract, as well as an inflammatory state, which damages the villous-crypt structure, the gastrointestinal barrier function and the microbiota.^6^^,^^7^ It has been shown that dietary supplementation with appropriate doses of vitamins, can adjust the intestinal function of weaned piglets and reduce the risk of inflammation and other diseases.^8–11^

Pantothenic acid, a form of water-soluble vitamin B5 (VB5), serves as a crucial raw material for the synthesis of coenzyme A (CoA) in cells. It plays a vital role in various metabolic pathways, such as glucose and fat metabolism, ensuring the maintenance of body homeostasis.^12^ While plants and microorganisms are capable of synthesizing VB5, animal cells lack this ability. Additionally, acetyl CoA can be converted into CoA.^13^ By increasing CoA levels and promoting glutathione synthesis, VB5 can enhance the inflammatory process and reduce oxidative stress.^14^^,^^15^ Moreover, VB5 significantly contributes to strengthening animals’ immunity and resistance against bacterial infections. It regulates the maturation of macrophages, promotes macrophage phagocytosis and restricts the growth of mycobacteria within macrophage cells.^16^ Activation of pro-inflammatory pathways, such as NF-κB and PI3K-AKT, leads to the production of cytokines in innate immune cells like macrophages, neutrophils and dendritic cells.^17^ Previous study found that VB5 is capable of activating different inflammatory signaling molecules (NF-κB, AKT, ERK and P38) and enhancing the expression of tumor necrosis factor-alpha (TNFα) and interleukin-6 (IL-6). These molecules play a crucial role as immune factors against tuberculosis.^18^ Furthermore, VB5 reduces triglyceride synthesis in the liver, promotes protein synthesis and conversion and facilitates the complete oxidation of fatty acids while decreasing ketone production.^19^ Inadequate intake of VB5 can lead to liver fat deposition and metabolic disorders.^20^ Deficiency in VB5 has been associated with growth failure, weight loss, dermatitis, dyslipidemia, neuropathy and adrenal disease.^21^ Therefore, supplementing animals with sufficient VB5 can improve their physiological condition.

The National Research Council (NRC) recommends adding 10 mg/kg of VB5 to the diet of weaned piglets weighing 7–11 kg. However, due to severe intestinal damage after weaning and reduced feed intake, piglets struggle to obtain enough feed to meet their VB5 needs. Therefore, it is necessary to increase the dose of VB5 in the diet of weaned piglets to meet their requirements, promote immune function and improve disease resistance. In this study, we hypothesized that the VB5 levels recommended by the NRC would lead to VB5 deficiency after weaning, which would worsen weaning stress. To address this, we added VB5 in higher amounts than the^22^ recommended levels (1 and 5 times) to the basal diet. This ensured a relatively high level of VB5 in the diet, which is crucial for normal growth and integrity of the piglet.^23–25^ A large number of studies have shown that supplementing the diet with VB5 can improve the growth performance of poultry, ruminants and growing pigs.^12^^,^^24^^,^^26^^,^^27^ The intestines of piglets grow faster than other body parts after weaning. Currently, there is limited data available on the effects of VB5 on the physicochemical aspects of weaned piglets’ intestines. Therefore, this study aimed to investigate the effects of different levels of VB5 on growth performance, intestinal development, short-chain fatty acid (SCFA) concentration and intestinal microbiota of weaned piglets. This may provide valuable insights into determining the optimal dosage of VB5 supplementation for weaned piglets.

## Materials and methods

### Animals and the experimental design

Twenty-one 21-day-old weaned piglets ([Large White × Landrace] × Duroc) were randomly assigned to three dietary treatments with seven replicate pens (one piglet per replicate pen) according to body weight (BW, 7.20 ± 1.11 kg) for 28-day period experiment, respectively. The L-VB5, control and H-VB5 groups were supplemented with an additional 0, 10 and 50 mg/kg of VB5 in the basal diet. The basal diet composition shown in Table 1 was designed to meet the nutrient requirements of weaned piglets.^22^ Phase I (day 0–14) basal diet contained 9.59 mg/kg VB5. On days 15, 16 and 17, the phase I feed was gradually replaced with the phase II ration at 25%, 50% and 75%, respectively, until all the feed of phase I was replaced. The phase II basal diet (days 18–28) contained VB5 of 9.18 mg/kg. During the trial, the piglets were kept in individual cages and were free to feed and drink. The trough was kept clean and the feed fresh to prevent the feed from getting moldy. Pig’s house was regularly cleaned and disinfected, and air circulation was regularly ventilated.

**Table 1.** Ingredient and chemical composition of piglet diets, as-fed basis.

Phase I		Phase II	
Ingredient	Content, %	Ingredient	Content, %
Corn	40.92	Corn	40.10
Extruded corn	20.00	Extruded corn	22.00
Soy protein concentrate	8.00	Soybean meal (43%)	20.50
Soybean meal (43%)	8.20	Fishmeal (63%)	4.00
Fishmeal (63%)	5.00	Whey powder	5.00
Whey powder	10.00	Glucose	3.00
Glucose	3.00	Soybean oil	1.50
Soybean oil	0.50	Limestone	0.58
Limestone	0.88	Dicalcium phosphate	0.88
Dicalcium phosphate	0.50	Choline chloride	0.10
Choline chloride	0.10	Antioxidants	0.05
Antioxidants	0.05	Acidifiers	0.50
Acidifiers	0.80	Salt	0.10
Salt	0.10	Mineral premix^a^	0.15
Mineral premix^a^	0.15	Vitamin premix^b^	0.50
Vitamin premix^b^	0.50	Lys (98%)	0.53
Lys (98%)	0.64	dl-Met	0.27
dl-Met	0.36	l-Thr	0.19
l-Thr	0.24	l-Trp	0.05
l-Trp	0.06		
Total	100	Total	100
Calculated nutrition level			
Net energy, Mcal/kg	2.49	Net energy, Mcal/kg	2.45
CP, %	18.61	CP, %	18.02
Ca, %	0.80	Ca, %	0.70
Available P, %	0.40	Available P, %	0.40
Lys^c^, %	1.35	Lys^c^, %	1.24
Met + Cys^c^, %	0.77	Met + Cys^c^, %	0.68
Thr^c^, %	0.79	Thr^c^, %	0.74
Trp^c^, %	0.22	Trp^c^, %	0.22

^a^Mineral premix: 100 mg/kg Zn (ZnSO_4_), 30 mg/kg Mn (MnSO_4_), 0.3 mg/kg Co (CoSO_4_), 150 mg/kg Fe (FeSO_4_), 25 mg/kg Cu (CuSO_4_), 0.3 mg/kg Se (Na_2_SeO_3_) and 0.5 mg/kg I (KIO_3_).

^b^Vitamin premix: 2200 IU/kg vitamin A, 17.5 μg/kg vitamin B12, 220 IU/kg vitamin D3, 16 IU/kg vitamin E, 0.5 mg/kg vitamin K3, 30 mg/kg niacin, 3.5 mg/kg riboflavin, 0.05 mg/kg biotin, 0.3 mg/kg folate acid, 1.0 mg/kg thiamin, 7 mg/kg pyridoxine, 4.0 mg/kg ethoxyquin.

^c^Stadardized ideal digestible.

### Sample collection

During the first week of the trial, one piglet in the control group died and the remaining piglets were sampled. The piglets were euthanized after fasting overnight. Following the measurement of the weight and total length of the small and large intestines from each piglet, the organ indexes (the ratio of the intestine’s total weight or total length to BW) were computed using the piglets’ 28-day BW. Approximately 2 cm samples of duodenum, jejunum, ileum, cecum and colon were taken and stored in a 4% formalin solution. The rest of duodenum, jejunum, ileum, cecum and colon were wrapped in tin foil, snap frozen in liquid nitrogen and then stored at −80 °C.^28^

### Growth performance measurement

Piglets were weighed at 0,14 and 28d of the trial to determine weight gain. Each pig’s feed consumption was also weighed daily to determine feed intake. The average daily gain (ADG), average daily feed intake (ADFI) and feed conversion ratio (FCR) for each pen were calculated at the end of the experiment.^29^ Record diarrhea per piglet per day to calculate diarrhea rate.^30^

### Intestinal histology and morphology analysis

Intestinal tissues were soaked in 4% formalin solution, sectioned using standard paraffin embedding methods and stained with hematoxylin and eosin (H&E). The height of villi and the depth of crypts were observed using a light microscope (Leica Imaging Systems Ltd, Cambridge, UK). Twenty intact villi and corresponding crypts were selected from each piglet and measured using Image-Pro Plus 6.0 software (Media Cybernetics, San Diego, CA). Goblet cells were mounted for Alcian blue-periodic Acid-shiff staining (Nanjing Jiancheng Bioengineering Institute, Nanjing, China), and they were counted in 20 intact villi and crypts.^28^^,^^30^

### Immunohistochemistry

Paraffin sections of jejunum with a thickness of 4 μm were dewaxed in xylene, then rehydrated with gradient alcohol and washed three times in phosphate buffer (pH =7.4). Block the endogenous peroxidase with 3% hydrogen peroxide. Then it was washed with PBS 3 times, then boiled twice in 0.01 mol/L and pH 6.0 citrate buffer for antigen repair. A total of 5% bovine serum albumin (Boster Biological Technology Co. Ltd, Wuhan, China) was reacted at 37 °C for 30 min. The sections were then incubated overnight with Ki67 antibody and Chga antibody (Cambridge, UK, Abcam;1:700 dilution) at 4 °C, and then washed with PBS 3 times. Then incubated with secondary antibodies (ZSGB-BIO, Beijing, China) at 37 °C for 1 h, washed with PBS 3 times, and positive cells were detected with diaminobenzidine kit (ZSGB-BIO). After anti-staining with hematoxylin, it was dehydrated by gradient alcohol. The samples were photographed at a magnification of 10 using a Leica DM 3000 microscope. For each piglet, 20 complete villi and crypts were collected.^31^

### Determination of SCFAs

Using a 10 mL centrifuge tube, weigh 1.000 g of fresh colon contents. Add 5 mL of distilled water, and centrifuge at 10000 × *g* for 15 min. In a 9:1 ratio, combine supernatant fluid with a 25% metaphosphoric acid solution. After centrifuging overnight, the solution was centrifuged in a 4 °C centrifuge (10000 × *g*) for 10 min and filtered through a membrane filter with 0.45 μm pore diameter and transferred to an injection vial. The SCFAs concentrations of samples were determined by using Agilent Technologies 7890B GC System (AGILENT, USA) with flame ionization detector on a DB-FFAP column (30 μm long × 250 μm diameter × 0.25 μm film thickness). Nitrogen was used as the carrier gas with a flow rate of 0.8 mL/min.^9^

### 16S rRNA high-throughput sequencing

Genomic DNA was extracted from colon stool samples, and then the V3–V4 region of 16s rRNA gene was amplified by PCR (universal bacterial primers 515F (5′-ACTCCTACGGGAGGCAGCAG-3′) and the reverse primer 806R (5′-GGACTACHVGGGTWTCTAAT-3′)). Samples were sequenced on the Illumina HiSeq2500 platform provided by Majorbio Bio-pharm Technology (Shanghai, China). Using a single-end sequencing method, a small fragment library was constructed for single-end sequencing. After distinguishing the samples, clustering and species classification analysis were conducted using the operational taxonomic units (similarity level of 97%) of the clean data. Then, the alpha and beta diversity measurements were done using QIIME (Version 1.9.1) software.^11^^,^^32^ Raw 16s rRNA data sequences have been uploaded to the SRA database (PRJNA915325).

### Statistical analysis

SPSS 22.0 statistical software (version 17.0; IBM Corp., Chicago, IL) was used for all statistical analyses. The data were examined for normal distribution and variance homogeneity. Use one-way analysis of variance if the means have a normal distribution; otherwise, use the chi-square test. Duncan’s multiple comparisons were used to estimate treatment differences, and values were presented as means ± SEM. *p* < .05 meant a significant difference, and.05 < *p* < .10 meant there is a trend of significant difference.

## Results

### Growth performance

During the 28-day trial period, there was no significant difference in initial BW, final weight, ADG, ADFI, FCR and diarrhea scores of piglets (Table 2). The results in Table 3 show that there were no differences in the length and weight of the small intestine and the weight of the large intestine in piglets; however, the length of the large intestine (*P* = 0.083) increased when the diet was supplemented with 50 mg/kg VB5. There were no differences in organ indices for small intestine weight/BW, small intestine length/BW, large intestine length/BW, small intestine weight/small intestine length and large intestine weight/large intestine length, whereas, increasing the dietary VB5 concentration significantly increased large intestine weight/BW (*p* = 0.016).

**Table 2.** The growth performance in piglet.^a^

Item	L-VB5	Control	H-VB5	SEM	*p* Value
BW, kg					
Initial BW	7.21	7.10	7.20	0.25	.984
14 days BW	8.01	8.21	8.18	0.33	.970
Final BW	12.81	12.90	13.06	0.54	.982
ADG, g					
Day 0–14	57.65	79.17	69.90	11.79	.780
Day 14–28	342.35	335.12	348.98	20.30	.967
ADFI, g/d					
Day 0–14	194.39	208.33	211.08	10.63	.801
Day 14–28	398.36	380.46	405.74	16.26	.832
FCR, g/g					
Day 0–14	3.06	2.42	2.73	0.21	.513
Day 14–28	1.83	1.72	1.74	0.06	.488
Diarrhea score					
Day 0–14	0.88	0.84	0.82	0.09	.966
Day 14–28	0.61	0.61	0.72	0.13	.936

^a^*n* = 7.

BW, body weight; ADG, average daily gain; ADFI, average daily feed intake; FCR, feed conversion rate.

**Table 3.** Effect of vitamin B5 on intestinal organ indices.^a^

Item	L-VB5	Control	H-VB5	SEM	*p* Value
Small intestine length, m	11.2	10.41	11.5	0.24	.195
Small intestine weight, g	552.77	534.81	589.91	24.05	.662
Large intestine length, m	2.13	2.02	2.31	0.05	.083
Large intestine weight, g	241.29	225	269.29	10.2	.212
Small intestine length/ BW, m/kg	0.9	0.82	0.91	0.04	.653
Small intestine weight/BW, g/kg	43.9	41.1	45.48	1.24	.379
Large intestine length/ BW, m/kg	0.17	0.16	0.18	0.01	.332
Large intestine weight/BW, g/kg	18.84^a^^b^	17.57^b^	20.85^a^	0.49	.016
Small intestine weight/small intestine length, g/m	49.32	50.96	51.21	1.68	.322
Large intestine weight/large intestine length, g/m	112.44	110.6	116.22	2.98	.758

^a^*n* = 7.

a, b Values in the same row with different superscript letters are significantly different (*p* < .05).

### Intestinal morphology and immunohistochemistry

There were significant differences in the ileal villus height (*p* = .016) and villus height/crypt depth (*p* = .011) between the treatments with the addition of VB5 and L-VB5 groups. The depth of the crypt in the cecum and colon was significantly increased in the H-VB5 group compared to the Control and the L-VB5 group (Table 4).

**Table 4.** Effects of vitamin B5 on intestinal morphology.^a^

Item	L-VB5	Control	H-VB5	SEM	*p* Value
Villus height, μm					
Duodenum, μm	284.21	308.69	281.95	8.32	.420
Jejunum, μm	293.14	294.11	265.53	8.03	.252
Ileum, μm	242.23^b^	304.86^a^	306.00^a^	11.34	.016
Crypt depth, μm					
Duodenum, μm	365.63	341.83	378.85	9.81	.353
Jejunum, μm	289.28	275.99	309.21	8.82	.325
Ileum, μm	305.77	281.13	281.62	9.13	.348
Cecum, μm	418.16^a^^b^	383.46^b^	469.21^a^	12.98	.017
Colon, μm	458.12^a^^b^	416.57^b^	513.05^a^	14.59	.020
Villus height: Crypt depth					
Duodenum	0.8	0.91	0.75	0.04	.240
Jejunum	0.99	1.07	0.88	0.05	.289
Ileum	0.90^b^	1.30^a^	1.21^a^	0.06	.011

^a^*n* = 7.

a, b Values in the same row with different superscript letters are significantly different (*p* < .05).

**Figure 1.**
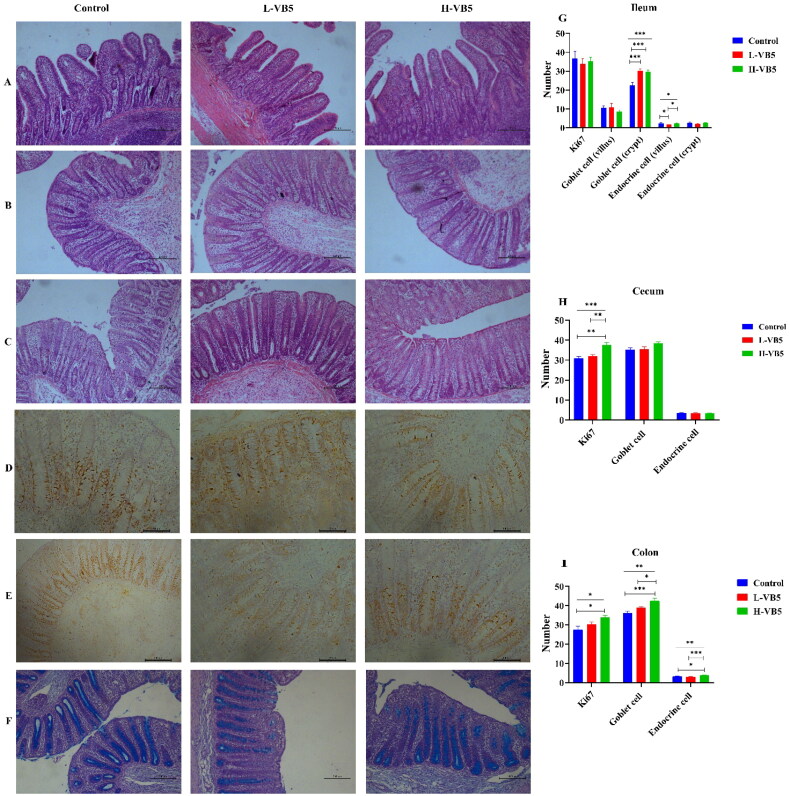
Intestinal morphological structure and immunohistochemical analysis of weaned piglets fed with different dietary concentrations of VB5 (magnification 100×). (A) ileum morphology. (B) Colon morphology. (C) Cecum morphology. (D) Immunohistochemistry staining with a Ki67 antibody in the colon. (E) Immunohistochemistry staining with a Ki67 antibody in the cecum. (F) Alcian blue-periodic acid-shiff staining in the colon. (G)Immunohistochemistry of the ileum. (H) Immunohistochemistry of the cecum. (I) Immunohistochemistry of the colon. *n* = 7. *p < .05, **p < .01, ***p < .001.

The number of goblet cells in the ileal crypt site was significantly increased in the L-VB5 and H-VB5 groups (*p* < .001), while the number of endocrine cells in the ileal villus site was significantly increased in the Control and H-VB5 groups (*p* = .029) as shown in Fig. 1G–I. The crypt depth (*p* = .017), the number of Ki67 positive cells (*p* = .001) and goblet cells (*P* = .08) of cecum were significantly increased in H-VB5 group, whereas VB5 did not affect cecal endocrine cells. The number of Ki67 positive cells (*p* = .023), goblet cells (*p* = .001) and endocrine cells (*p* = .001) of colon were significantly increased in H-VB5 group.

### Microbial assessment in colon contents

**Figure 2.**
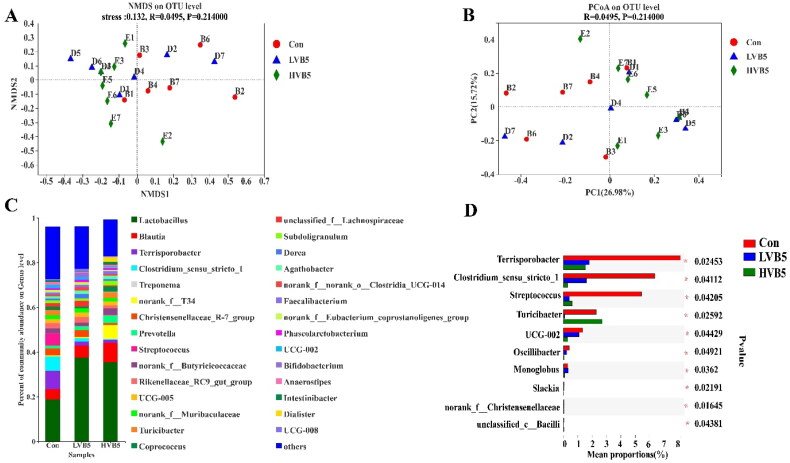
Effect of vitamin B5 on the composition of colonic bacterial. (A) NMDS plot of functional profiles among groups. (B) PCoA plot of functional profiles among groups. (C) Colon bacterial communities at the genus level. (D) Colonic differential bacteria at the genus level. *n* = 7. *p < .05, **p < .01, ***p < .001.

Colonic microbial alpha diversity indices showed no differences in Sobs, ACE, Shannon, Simpson and Chao1 indices for the three groups, as shown in Table 5. The principal co-ordinates analysis (PcoA) and non-metric multidimensional scaling (NMDS) analysis (Fig. 2B and C) showed no difference in the composition of the three groups at the OUT level.

**Table 5.** Alpha diversity index.^a^

Items	L-VB5	Control	H-VB5	SEM	*p* Value
Sobs	446.71	456.00	381	22.04	.330
ACE	509.84	531.40	452.49	23.63	.392
Shannon	3.67	3.99	3.42	0.11	.291
Simpson	0.11	0.06	0.11	0.14	.259
Chao1	520.33	533.98	451.85	23.42	.321

^a^*n* = 7.

At the genus level, the top five dominant species were *Lactobacillus*, *Blautia*, *Terrisporobacter*, *Clostridium_sensu_stricto_1* and *Treponema* (Fig. 2C). The top 10 statistically different species at the genus level are shown in Fig. 2D. *Terrisporobacter*, *Clostridium_sensu_stricto_1*, *Streptococcus* and *UCG-002* in the H-VB5 group had significantly lower abundance (*p* < .05) compared to the Control group. *Turicibacter* in the Control and H-VB5 groups showed a significant increase in abundance compared to the L-VB5 group (*p* = .025).

### Effect of VB5 on metabolic function of microbiota

**Figure 3.**
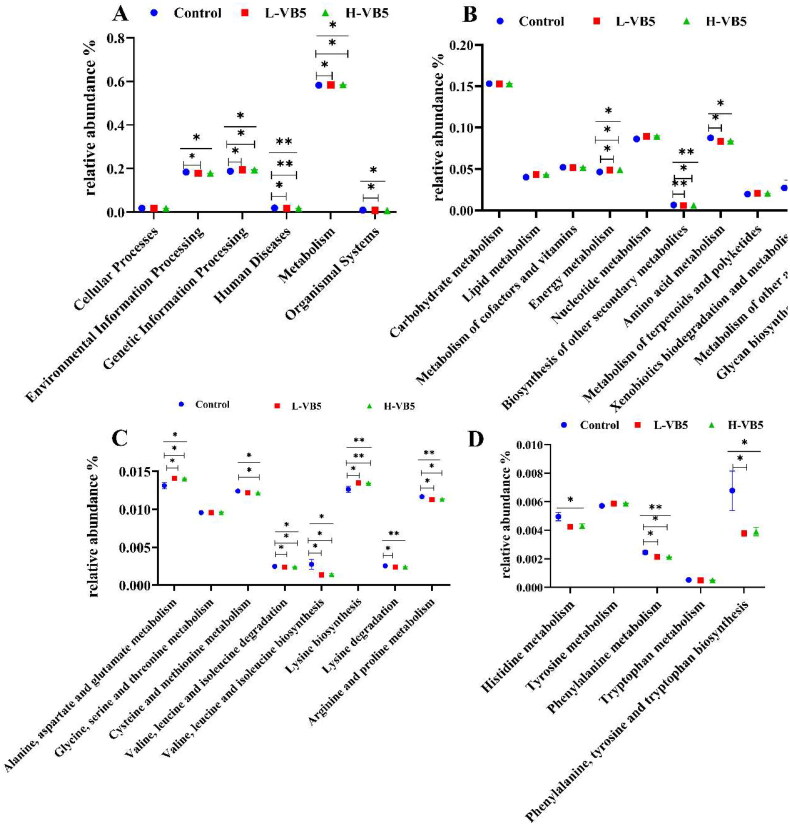
Abundances of KEGG pathways in the functional prediction by Tax4Fun. (A) Prediction of colonic microbial function. (B) Metabolic function annotation map. (C) and (D) Amino acid metabolism. *n* = 7. *p < .05, **p < .01, ***p < .001.

The effects of additional VB5 addition on colonic bacteria were focused on six aspects, and the most significant effect on metabolism, which include energy metabolism, biosynthesis of other secondary metabolites, amino acid metabolism, xenobiotics biodegradation and metabolism, metabolism of other amino acids and glycan biosynthesis and metabolism (Fig. 3A and B). The addition of VB5 enhanced the alanine, aspartic acid and glutamic acid metabolism; cysteine and methionine metabolism; valine, leucine and isoleucine degradation; valine, leucine and isoleucine biosynthesis; lysine biosynthesis; lysine degradation; arginine and proline metabolism; histidine metabolism; phenylalanine metabolism and phenylalanine, tyrosine and tryptophan biosynthesis (Fig. 3C and D).

### Correlation analysis of gut morphology and microbes

**Figure 4.**
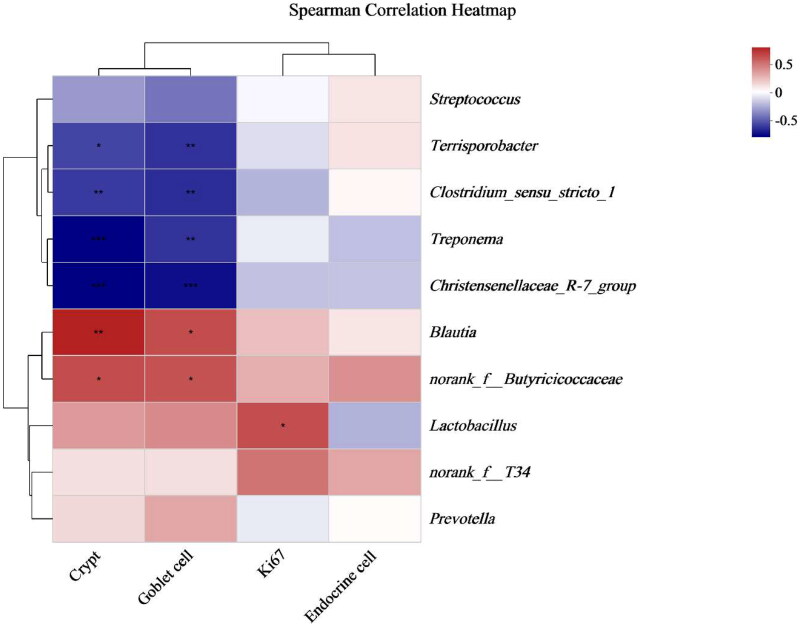
Spearman correlation analysis of gut morphology and microbes (genus level). *p < .05, **p < .01, ***p < .001.

Spearman correlation analysis was conducted between the top 10 bacterial genera, and the depth of the colonic crypts, ki67-positive cells, endocrine cells and goblet cells are directly reflected by a heatmap (Fig. 4). The results indicated that *Blautiu* and *norank_f__Butyricicoccaceae* were positively correlated with crypt depth and goblet cells. *Terrisporobacter*, *Clostridium_sensu_stricto_1*, *Treponema* and *Christensenellaceae_R-7_group* were negatively correlated with crypt depth and goblet. *Lactobacillus* was positively correlated with ki67.

### Effect of VB5 supplementation on the SCFAs concentration

There were no differences in the levels of acetic acid, propionic acid and valeric acid in the three groups; however, the concentration of isobutyric acid (*p* = .007) and isovaleric acid (*p* = .034) significantly decreased with increasing VB5 levels, and the content of butyric acid was decreased with increasing VB5 levels (*p* = .073) as shown in Table 6.

**Table 6.** Effect of VB5 on colonic SCFAs.^a^

Item, μg/g	L-VB5	Control	H-VB5	SEM	*p* Value
Acetic acid	3451.79	3018.71	3176.17	135.50	.470
Propionic acid	1257.29	1327.61	1118.37	79.31	.577
Isobutyric acid	142.34^a^	161.97^a^	82.70^b^	12.29	.007
Butyric acid	688.77	611.99	404.49	55.10	.073
Isovaleric acid	201.80^a^	92.62^b^	87.62^b^	21.64	.034
Valeric acid	208.95	97.43	118.58	29.21	.281

^a^*n* = 7.

a, b Values in the same row with different superscript letters are significantly different (*p* < .05).

## Discussion

Previous report had shown that there was no significant effect of six different levels of pantothenic acid on weight gain rate, feed intake and blood biochemical properties of piglets,^33^ In addition, Groesbeck et al. have reported no effect of increasing the amount of pantothenic acid on ADG, ADFI and FCR of test pigs.^24^ In the present study, the addition of 0, 10 and 50 mg/kg VB5 did not affect the growth performance of piglets, which is consistent with Groesbeck’s and Krehl’s results. Weaning stress has been linked to changes in intestinal morphology and structure, including shortening of intestinal length, villus atrophy and crypt hyperplasia, which in turn reduces the absorption rate of piglets.^30^^,^^34^ The magnitude of villus height/crypt depth can represent the level of intestinal digestive and absorptive capacity.^35^ Piglets absorb nutrients through the villus site, and an increase in villus height indicates that more nutrients are in contact with the villus, allowing more nutrients to be absorbed.^36^ The villus height was higher in the Control, H-VB5 group than in the L-VB5 group in this experiment, indicating that VB5 effectively protected the intestinal villus and reduced the damage to the intestine caused by weaning stress. Furthermore, VB5 increases the ratio of villus height/crypt depth, most likely due to the high concentration of VB5 increased the rate of migration of crypt stem cells from the bottom to the top villus, which has a positive effect on intestinal repair.^37^ Organ index can indicate the effectiveness of organ function,^9^ and the impact of nutrition on intestine development can be determined by intestinal weight and length.^38^ The addition of 50 mg/kg VB5 significantly increased the proportion of large intestine weight/BW ratio in piglets, and the large intestine length showed an increasing trend. The rapid proliferation and differentiation of intestinal epithelial cells is a prerequisite for normal intestinal growth.^39^ In this study, the crypt depth of the cecum and colon was significantly increased in the H-VB5 group. Ki67-positive proliferating cells were significantly increased in the colon and cecum. The goblet and endocrine cells were significantly increased in the colon. In addition, the goblet cells in the cecum showed an increasing trend, which may explain the increased weight of intestinal tissues in the H-VB5 group. This shows that VB5 supplementation has a positive effect on the development of large intestine in weaned piglets.

The villus mediates nutrient absorption and acts as a barrier in the intestinal lumen against antigens, pathogens and toxins.^40^ To ensure the function of the small intestine and to maintain a continuous and effective barrier function in a harsh intestinal environment, intestinal villus epithelial cells renew rapidly through cell proliferation and specific differentiation.^41^ The majority of ki67 cells are found in the intestinal crypt, and counting ki67 cells is a common way to measure the proliferation of intestinal epithelial cells.^42^ This study showed that VB5 did not affect the number of ileal ki67-positive cells. Previous studies have shown that the decrease in VH is either the result of increased cell loss or decreased crypt cell proliferation.^37^^,^^43^ The height of the ileal villus was significantly higher in the Control and H-VB5 groups than in the L-VB5 group, likely because VB5 protects the intestinal villus and reduces the rate of epithelial cell loss from the villus.^37^ The H-VB5 group showed a significant increase in ki67-positive proliferating cells in the colon and cecum, as well as an increase in crypt depth and an increase in epithelial cells. These results indicate that high-dose VB5 alters intestinal morphology primarily by promoting continuous proliferation and differentiation of crypt in the colon and cecum to produce new enterocytes.

By the creation of energy by immune cells, VB5 contributes to the maintenance of host immunity.^44^ Goblet cells, which are responsible for secreting and synthesizing mucus, are found in the mucosal epithelium. Goblet cells are required for the intestinal environment to be stable.^45^ The L-VB5 group had significantly more goblet cells in the ileal crypts than the control group in this study, likely to reduce the intestinal damage caused by microbial diseases from early weaning by secreting large amounts of mucus.^37^ Pantothenic acid is predominantly present in cells in the active form of CoA^46^ and acetyl CoA synthesizes acetylcholine,^47^^,^^48^ which is an effective inducer of intestinal mucus secretion and increases the rate of mucus secretion.^49^ Acetylcholine mainly targets goblet cells in the small intestinal crypts.^49^ The significant increase in the number of goblet cells in the crypt of the H-VB5 group could be attributed to the increase in CoA in the body caused by the addition of 50 mg/kg of VB5, which promotes the differentiation of goblet cells and the secretion of mucus, thereby protecting the intestine. The addition of 50 mg/kg of VB5 significantly increased the number of both colonic and the cecum goblet cells also showed an elevated trend, indicating that VB5 improved the chemical barrier of the intestine, boosted the immunity of the body, and enhanced the disease resistance. Many gut hormones are produced by enteroendocrine cells, and they play critical roles in the control of hunger, insulin secretion, and food digestion and absorption.^50^ In this investigation, endocrine cells increased significantly in the H-VB5 and control group. This indicates that VB5 promotes the differentiation of intestinal enteroendocrine cells.

It is generally recognized that SCFAs play a crucial role in maintaining the balance of the intestinal microbiota and the function of the intestinal epithelium.^51^ The concentration of isobutyric acid and isovaleric acid is proportional to the amount of undigested protein in the intestine, and their primary source is the fermentation of undigested protein flowing from the ileum into the hindgut by hindgut microorganisms.^51^ In this study, the VB5-containing diet may improve ileum intestinal morphology and nutrient digestion, thereby improving the feed utilization of piglets.^52^ This diet may cause more branched-chain amino acid digestion, resulting in a significant decrease in isobutyric acid and isovaleric acid concentrations in the H-VB5 group. SCFAs play a crucial role in maintaining the structural integrity and function of colon epithelium.^53^ SCFA can effectively stimulate the proliferation of intestinal epithelial cells by serving as an energy substrate, generally, colon epithelial cells primarily utilize butyrate.^9^ In this experiment, the concentrations of butyric acid, isobutyric acid and isovaleric acid were found to be the lowest in the colon contents of the 50 mg/kg VB5 group. Additionally, the 50 mg/kg VB5 group exhibited improved intestinal morphology, cell proliferation and differentiation. These findings suggest that VB5 may enhance the utilization of SCFAs by colon epithelial cells, thereby promoting colon development, which may explain why the highest number of proliferating and differentiated cells in the H-VB5 group. The Spearman correlation analysis of gut morphology and microbes showed that the abundance of *Terrisporobacter* and *Clostridium_sensu_stricto_1* was negatively correlated with the crypt depth and goblet cells. The content of *Terrisporobacter* and *Clostridium_sensu_stricto_1* was significantly decreased in the H-VB5 group, indicating that VB5 may influence the colon’s morphology and structure by modifying the number of microorganisms present there.

The intestinal microbiota, as a critical component in metabolic control, plays an important role in catabolism and nutrient absorption.^54^ In our study, the effects of VB5 supplementation on intestinal microflora are variable. At the genus level, the addition of 50 mg/kg VB5 significantly increased the abundance of beneficial bacteria (*Turicibacter*)^55^ and reduced the abundance of harmful bacteria^56^ (*Terrisporobacter*, *Clostridium_sensu_stricto_1*, *Streptococcus*) and *UCG-002* compared with the Control group abundance. *Turicibacter* plays a few beneficial roles in immune interactions within the porcine microbiome, thereby promoting growth performance.^57^ *Clostridium_sensu_stricto_1*, a butyrate producer, is generally considered pathogenic and is associated with inflammation and neonatal diarrhea, as well as causing colonic mucosal damage that induces inflammation.^58^ In contrast, *Terrisporobacter,* on the other hand, is an emerging anaerobic pathogen that produces toxins such as trimethylamine-N-oxide, which is associated with oxidative stress and inflammation in the intestine of weaned pigs.^56^^,^^59^ *Streptococcus* is a pathogenic microorganism responsible for various diseases in pigs.^60^ *UCG-002* are beneficial bacteria that promote fiber breakdown and digestion and are involved in the regulation of enteritis and reducing intestinal inflammation.^61^ In addition, they can ferment lacto-oligosaccharides to produce SCFA for microbial regulation and epithelial development. The reduced abundance of *UCG-002* in the H-VB5 group may be due to the addition of 50 mg/kg VB5, which increased villus height of the ileum and endocrine cells of the colon. Therefore, increasing in intestinal absorptive capacity, a decrease in carbohydrate reaching the posterior end of the colon, and a positive correlation between *UCG-002* abundance and the concentration of isobutyric acid. This may also account for the lowest isobutyric acid concentration in the colonic contents of the H-VB5 group. In conclusion, the altered intestinal microbiota induced by the additional 50 mg/kg VB5 treatment in the basal diet could result in improved intestinal health and stabilization of the immune status and intestinal environment. Further Tax4Fun analysis of microbiota function predictions revealed that the addition of VB5 significantly altered amino acid metabolism-related enrichment pathways. Many amino acids are believed to be required for the maintenance of intestinal health and to regulate intestinal immunity.^62^ All of these findings suggest that the addition of 50 mg/kg VB5 improves the health of the piglet gut.

## Conclusions

The results of this study indicate that the inclusion of 50 mg/kg VB5 in the diet of weaned piglets can enhance the length and weight of the large intestine, as well as improve the intestinal morphology of the ileum, cecum and colon. Additionally, it was observed that VB5 influences colon development by impacting the abundance of colon microbial populations and the content of SCFAs. Furthermore, the supplementation of 50 mg/kg VB5 led to an increase in the abundance of beneficial bacteria (*Turicibacter*) in the pig colon. These findings provide a scientific basis for the use of VB5 to improve the health of weaned piglets by regulating organ index, intestinal morphology, intestinal flora and SCFA concentration.

## Animal welfare statement

All animal experimental procedures were performed according to protocols approved by the Animal Care Advisory Committee of Hunan Normal University, Changsha, Hunan, China.
